# A Nomogram Combining MRI Multisequence Radiomics and Clinical Factors for Predicting Recurrence of High-Grade Serous Ovarian Carcinoma

**DOI:** 10.1155/2022/1716268

**Published:** 2022-05-04

**Authors:** Cuiping Li, Hongfei Wang, Yulan Chen, Mengshi Fang, Chao Zhu, Yankun Gao, Jianying Li, Jiangning Dong, Xingwang Wu

**Affiliations:** ^1^Department of Radiology, The First Affiliated Hospital of Anhui Medical University, Hefei 230022, China; ^2^Department of Radiology, The First Affiliated Hospital, University of Science and Technology of China, Anhui Provincial Cancer Hospital, Hefei 230031, China; ^3^Department of Radiotherapy, The First Affiliated Hospital, University of Science and Technology of China, Anhui Provincial Cancer Hospital, Hefei 230031, China; ^4^CT Research Center, GE Healthcare, China

## Abstract

**Objective:**

To develop a combined nomogram based on preoperative multimodal magnetic resonance imaging (mMRI) and clinical information for predicting recurrence in patients with high-grade serous ovarian carcinoma (HGSOC).

**Methods:**

This retrospective study enrolled 141 patients with clinicopathologically confirmed HGSOC, including 65 patients with recurrence and 76 without recurrence. Radiomics features were extracted from the mMRI images (FS-T2WI, DWI, and T1WI+C). L1 regularization-based least absolute shrinkage and selection operator (LASSO) regression was performed to select radiomics features. A multivariate logistic regression analysis was used to build the classification models. A nomogram was established by incorporating clinical risk factors and radiomics Radscores. The area under the curve (AUC) of receiver operating characteristics, accuracy, and calibration curves were assessed to evaluate the performance of classification models and nomograms in discriminating recurrence. Kaplan-Meier survival analysis was used to evaluate the associations between the Radscore or clinical factors and disease-free survival (DFS).

**Results:**

One clinical factor and seven radiomics signatures were ultimately selected to establish the predictive model for this study. The AUCs for identifying recurrence in the training and validation cohorts were 0.76 (0.68, 0.84) and 0.67 (0.53, 0.81) with the clinical model, 0.78 (0.71, 0.86) and 0.74 (0.61, 0.86) with the multiradiomics model, and 0.83 (0.77, 0.90) and 0.78 (0.65, 0.90) with the combined nomogram, respectively. The DFS was significantly shorter in the high-risk group than in the low-risk group.

**Conclusion:**

By incorporating radiomics Radscores and clinical factors, we created a radiomics nomogram to preoperatively identify patients with HGSOC who have a high risk of recurrence, which may serve as a potential tool to guide personalized treatment.

## 1. Introduction

In an age of rapid technological and medical advances, ovarian cancer (OC) diagnosis still poses a daunting challenge to doctors. High-grade serous ovarian carcinoma (HGSOC) is the most common histological subtype in the clinic, with insidious symptoms and high aggressiveness. At the time of initial diagnosis, approximately 70% of ovarian cancer patients are already in an advanced stage [1, 2], resulting in a poor prognosis. The optimal treatment of patients with HGSOC consists of primary debulking surgery (PDS) followed by platinum-based adjuvant chemotherapy or neoadjuvant chemotherapy (NACT) combined with interval debulking surgery (IDS) [3, 4]. Approximately 80% of patients can benefit from this therapeutic strategy, which is associated with a high rate of complete clinical remission [5]. However, approximately 80% of patients with advanced HGSOC will experience tumour recurrence with less than 3 years of recurrence-free survival (RFS) [2, 6]. The survival after recurrence is poor, regardless of early or advanced stage disease [7]. In addition to late detection, preoperative hormone levels, treatment strategies, and tumour residual state are also important factors that are strongly associated with recurrence. However, to date, there are no reliable prognostic biomarkers for clinical application [8]. Hence, developing a method to detect the risk of recurrence of HGSOC is of great importance for prolonging RFS and improving the prognosis of patients.

Conventional magnetic resonance imaging (cMRI) plays a significant role in the diagnosis of pelvic diseases due to its high soft-tissue resolution [9, 10]. It can clearly reveal the lesion morphology, characteristics (edema, hemorrhage, fibrosis, and so on), the relationship between the lesion and surrounding tissues, and even the status of lymph nodes (metastatic or inflammatory). The novel functional imaging technology of diffusion-weighted imaging (DWI), which can well reflect the differences in cell density and reveal the diffusion information of biological tissues [11–13], has been widely used in clinical practice to distinguish benign and malignant tumours, tumour grade, and differentiation [11, 14–16].

Radiomics analysis is a postprocessing method for extracting information by quantifying the spatial distribution of pixels or voxels with different grey intensities and counting the variables, that is, calculating and extracting texture features based on the texture matrix of the images [17–19]. Considering the large size and complexity of ovarian masses, radiomics analysis based on the whole tumour could more accurately reflect the heterogeneity of HGSOCs by quantifying complex parameter distributions and provide more accurate information for clinical practice [11, 20]. Previous researchers have found that FIGO stage, histological grade, subtype, and tumour residue are important risk factors for predicting postoperative recurrence. Previous studies included those based on the preoperative clinical risk factors for predicting the 3-year and 5-year survival rates of patients with epithelial ovarian cancer (EOC) [21], studies based on an analysis of CT images to identify HGSOC risk of recurrence markers [6], studies based on CT radiomics for predicting early recurrence of high-grade serous ovarian cancer [22], and a set of radiomics models based on MRI for predicting recurrence of patients with advanced high-grade serous ovarian cancer risk [5]. These studies have achieved certain progress but are not comprehensive. Our study is aimed at developing and evaluating a nomogram combining a radiomics model based on preoperative multimodal magnetic resonance imaging (mMRI) and clinical information for predicting recurrence in patients with HGSOC to achieve precise and individualized diagnosis and treatment.

## 2. Materials and Methods

### 2.1. Patients

The institutional review board of Anhui Provincial Cancer Hospital (West Branch of The First Affiliated Hospital, University of Science and Technology of China), approved this retrospective study, and the requirement for informed consent was waived. Our study was conducted in accordance with the Declaration of Helsinki.

We retrospectively reviewed the clinical records and imaging results of 191 consecutive patients with surgically and pathologically proven HGSOC who underwent pelvic MRI examination at Anhui Provincial Cancer Hospital between July 2014 and December 2019. Patients were excluded from the study if they were lost to follow-up within less than 18 months (*n* = 44), had few solid components (*n* = 3), or had incomplete pathological reports (*n* = 3). Finally, 141 patients with HGSOC were enrolled in the present study. Various clinical indicators of the patients were obtained through the medical record system, including age at diagnosis, preoperative carbohydrate antigen 125 (CA125), and human epididymis protein 4 (HE4) levels. The median age of the patients was 54 years (age range, 41-78 years). The tumours were staged according to the 2014 International Federation of Gynaecology and Obstetrics (FIGO) guidelines [21]. The process of patient selection is illustrated in Figure S1.

### 2.2. MRI Protocol

MRI examination was performed using an MR system (GE Signa HDXT 3.0 T MRI scanner, GE Healthcare, USA) equipped with an 8-channel phased-array abdominal coil. All patients received an intramuscular injection of 15 mg hyoscine butylbromide 30 minutes before the MRI scan to prevent gastrointestinal motility. The bladder was kept approximately half-filled to improve lesion visibility without changing the anatomy. Patients were placed in the supine position and were breathing freely during the acquisition.

The routine pelvic MRI protocol consisted of the following sequences: axial T1-weighted imaging (T1WI), axial/sagittal T2-weighted imaging (T2WI), axial fat-suppressed T2-weighted imaging (FS-T2WI), diffusion-weighted imaging (DWI) (*b* value = 0, 1,000 s/mm^2^), and multiple phases of contrast-enhanced (LAVA-FLEX) MRI. When scanning the axial images, the transverse plane was perpendicular to the long axis of the uterine body, and for the sagittal images, the longitudinal plane was parallel to the main body of the uterus. Contrast-enhanced T1-weighted imaging (T1WI+C) sequences were acquired at the arterial, venous, and delayed phases of contrast medium enhancement in the axial planes, which were acquired at 25, 60, and 120 s after the intravenous injection of 0.1 mmol/kg gadodiamide (Omniscan, GE Healthcare) using an Ulrich power injector. The details of the scanning sequences and parameters are shown in Table S1.

### 2.3. MRI Image Analysis

**Figure 1:**
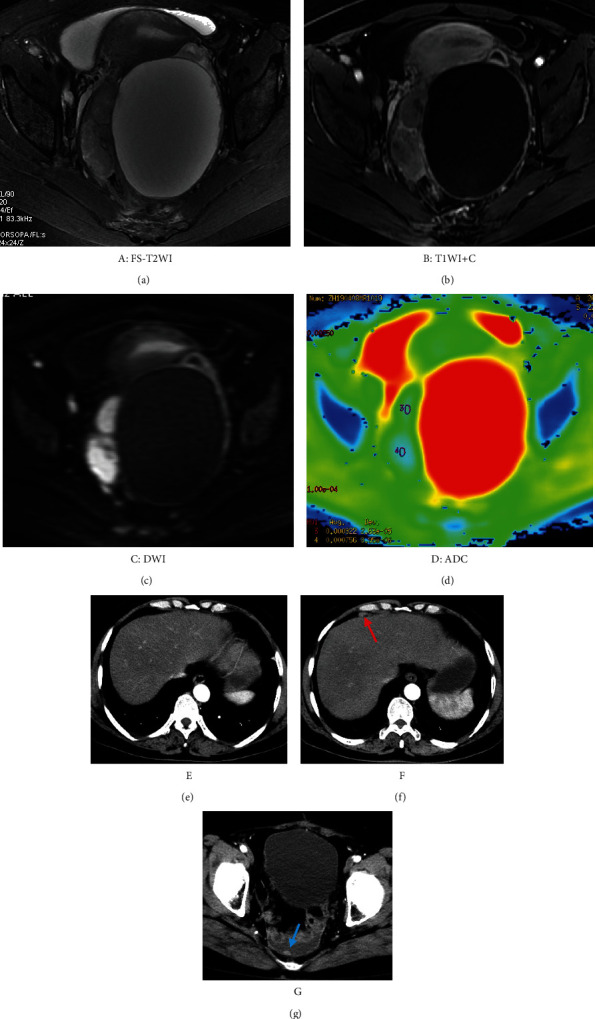
(a–d) A 54-year-old female was diagnosed with bilateral ovarian high-grade serous carcinoma (HGSOC, FIGO IIIC) by postoperative pathology on April 18, 2019. Postoperative chemotherapy was performed six times with TC regimen [docetaxel (75 mg/m^2^) + carboplatin (AUC = 5)]. (e) Reexamination of chest+abdominal CT and pelvic MRI on January 20, 2020. No clear recurrent diseases were found. (f, g) On April 25, 2021, the CA125 was elevated, and CT examination revealed thickening of the abdominal peritoneum with multiple nodules (red and blue arrow) and pelvic effusion, so recurrence was considered. DFS was 24 months.

Two radiologists with more than 10 years of experience in gynecological imaging analysed the images without knowing the pathological results of these patients and reached a consensus (Figure 1). Using a GE ADW 4.6 postprocessing workstation, the DWI images of the tumour layer with *b* = 1000 s/mm^2^ were analysed, and the ADC values were calculated. The measurement was repeated three times, and then the average value was obtained. When sketching the region of interest (ROI), the T2WI and T1WI+C images were referenced to determine the tumour boundary, and the mucus, necrosis, cystic change, and bleeding areas were avoided.

### 2.4. MRI Image Segmentation and Radiomics Feature Extraction

Manual segmentation was performed based on FS-T2WI, DWI, and T1WI+C sequences by using ITK-SNAP software (version 3.8.0, http://www.itksnap.org). The ROI of each tumour was manually contoured along the boundary of the tumour, and the volume of interest (VOI) was constructed by ROI interpolation for each slice. The interobserver reproducibility was initially analysed using 30 randomly chosen images for the VOI by the 2 radiologists mentioned above. Intraclass correlation coefficients (ICCs) were used to evaluate the interobserver agreement of the measurement of radiomics features (ICC > 0.75 was indicative of almost perfect agreement).

To reduce the discrepancies between imaging parameters, several preprocessing steps of the MR images were applied before the process of radiomics feature extraction. All images were resampled to a voxel size of 1 × 1 × 1 mm^3^ using B-spline interpolation. Each MRI scan was normalized to obtain a standard normal distribution of image intensities. Radiomics features were extracted from 3 types of multisequence MR images (FS-T2WI, DWI, and T1WI+C) for each lesion using PyRadiomics software (http://pyradiomics.readthedocs.io/en/latest/index.html), which can automatically obtain the histogram parameters for the whole solid tumour VOI. Seven classes of 1316 radiomics features were extracted: shape features, first-order features, grey-level cooccurrence matrix (GLCM) features, grey-level run-length matrix (GLRLM) features, grey-level size zone matrix (GLSZM) features, neighbourhood grey-tone difference matrix (NGTDM) features, and grey-level dependence matrix (GLDM) features (Table S2). A detailed description of the radiomics image preprocessing is shown in Figure S2.

### 2.5. Data Preprocessing

The dataset was randomly assigned in a 7 : 3 ratio to either the training cohort or the validation cohort. All cases in the training cohort were used to train the predictive model, while cases in the validation cohorts were used to independently evaluate the model's performance.

Variables with zero variance were excluded from the analyses. Then, the missing values and outlier values were replaced by the median. Finally, the data were standardized.

### 2.6. Feature Selection and Classifier Modelling

First, features with ICCs > 0.75 were retained. Second, feature selection was performed by using univariate logistic analysis (Correlation-xx) and LASSO with a stepwise selection method. The Radscore value was calculated as (radiomics signature × coefficient) + intercept. Finally, a logistics-based Radscore model was built based on the established optimal feature subsets of the training cohort.

**Figure 2:**
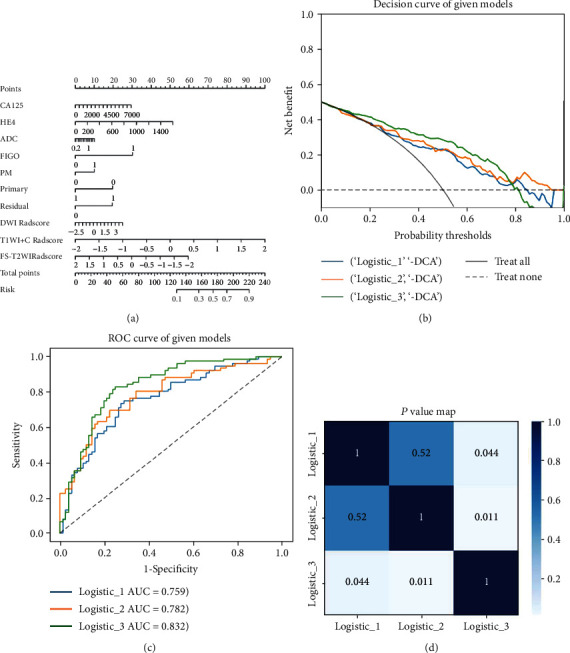
(a) Nomogram based on radiomics signatures and clinical factors. In the nomogram, a vertical line was made according to each parameter to determine the corresponding value of points. The total points were the sum of the three points above. Then, a vertical line was made according to the value of the total points to predict the recurrence probability of HGSOC. (b–d) Model's performance assessment and comparison. (b) Decision curve analysis of radiomics signature, clinical model, and nomogram, respectively. (c) Receiver operating characteristic curve analysis in the validation cohorts. (d) Delong test for the given models.

After that, we incorporated the radiomics Radscore and clinical risk factors into a nomogram using multivariable logistic regression analysis (Figure 2).

Receiver operating characteristic (ROC) curves were generated to determine the performance of the models, and the accuracy, sensitivity, specificity, and area under the curve (AUC) of using these models for predicting tumour recurrence were calculated. The calibration curves were assessed to evaluate the model's performance in predicting recurrence. The differences for each model were compared using the DeLong test method (Figure 2).

### 2.7. Follow-Up and Clinical Endpoint

All enrolled patients were required to undergo clinical follow-up visits every 2-4 months for 2 years, every 3-6 months for 3 years, and then annually after 5 years, as suggested by the National Comprehensive Cancer Network (NCCN) guidelines. Tumour markers, a physical examination including a pelvic examination, and a B-mode ultrasound examination (abdomen and pelvis) were conducted at each follow-up visit. Patients were also required to undergo annual imaging examinations, such as CT, MRI, and PET/CT. During the follow-up period, if the CA125 value was dynamically elevated, a CT/MRI or PET/CT examination of the chest, abdomen, pelvis, or a combination was required to determine if recurrent disease was present.

We defined DFS as the time interval from the date of surgery or NACT to the first date of disease recurrence, and we used the date of the last follow-up to confirm no evidence of recurrence. Recurrence was determined by histologic results or by the combination of imaging evidence and serum CA125 levels according to the Gynaecologic Cancer Intergroup (GCIG) criteria. Patients without recurrent disease were followed up for at least 18 months.

### 2.8. Statistical Analysis

Commercial software (SPSS 22.0, IBM Corporation, Armonk NY, USA) was used for the statistical analysis. We tested whether the numerical variables were normally distributed by using a one-sample Kolmogorov–Smirnov test. Data with a normal distribution are expressed as the mean ± standard deviation (*M* ± SD), while nonnormally distributed data are expressed as the median (interquartile range (IQR), 25th and 75th percentiles). An independent sample *t*-test was used for data conforming to a normal distribution, while a Mann–Whitney *U* test (a nonparametric rank-sum test) was used for data conforming to a nonnormal distribution. The chi-square test was used for unordered categorical variables. To measure the associations between the risk scores derived from the prediction models and the RFS status of patients, the early recurrence rate was obtained using Kaplan–Meier survival analysis and Cox regression analysis. *P* < 0.05 was considered statistically significant.

## 3. Results

### 3.1. Analysis of Clinical Factors

The basic clinical characteristics of all patients in our dataset are summarized in Table 1. Of the 141 patients with HGSOC, 65 patients developed recurrent disease during the follow-up period, and the remaining 76 patients were censored with at least 18 months of follow-up. The median DFS was 27 months (range, 2-53 months). The pretreatment CA125 and HE4 indicators in the recurrence group were significantly higher than those in the nonrecurrence group (817.7 (564.5, 2,309.0) vs. 480.1 (220.4, 1062.3) *P* = 0.001, 499.1 (271.2, 1059.5) vs. 293.5 (175.4, 589.5) *P* = 0.001, respectively). The ADC value in the recurrence group was 0.83 (0.73, 0.92), which was significantly lower than that in the nonrecurrence group (0.90 (0.77. 0.99), *P* = 0.011). A total of 63.8% (90/141) of patients received PDS and postoperative adjuvant chemotherapy, and 36.2% (51/141) of patients received NACT due to late staging or large tumours. After surgery, R0 and R1 resection was achieved in 52.5% (74/141) and 47.5% (67/141) of patients, respectively. The majority (114/141, 80.9%; 122/141, 86.5%) of patients had peritoneal metastasis and FIGO III-IV stage disease. The multivariate analysis showed that the residual tumour status (R0/R1) was an independent predictor of tumour recurrence (OR = 4.51; 95%CI = 1.252-16.216; *P* = 0.021) (Table S3). Clinical statistics for the training cohort and validation cohort are provided in the supplementary materials (Table S4).

### 3.2. Radiomics Models and Model Comparisons

In total, 1316 radiomics features were extracted from each VOI of the three sequences. The ICC values ranged from 0.771 to 0.988, showing great interobserver agreement. From the initial feature pool, the T1WI+C-based model selected two features, including wavelet LHL glcm correlation and original shape flatness; the DWI-based model selected two features, including wavelet LLH first-order kurtosis and original shape flatness; and the FS-T2WI-based model selected three features, including wavelet LLH glcm idn, original glcm mcc, and original glszm small area low grey level emphasis (Table S5). Although some features had low discrimination power in distinguishing between recurrence and nonrecurrence cases (e.g., original glcm mcc and original glszm small area low grey level emphasis based on FS-T2WI), these features may provide supplementary information when mixed with other features.

Based on the above features, radiomics models of single sequence and multisequence combinations were established.  Table 2 lists the AUC values, accuracies, and corresponding 95% CIs of different models. In the training cohort, the AUC for the model using DWI, T1WI+C, FS-T2WI, and a fusion of three sequence features was 0.76 (0.68, 0.83), 0.73 (0.64, 0.80), 0.72 (0.64, 0.80), and 0.78 (0.71, 0.86), respectively. By incorporating both radiomics and clinical features in the combined model, its AUC improved to 0.83 (95% CI, 0.77, 0.90). Figure S3 shows the ROC, calibration curves and DCA curves of the different models in the validation cohort.

### 3.3. Analysis of Recurrence-Free Survival

**Figure 3:**
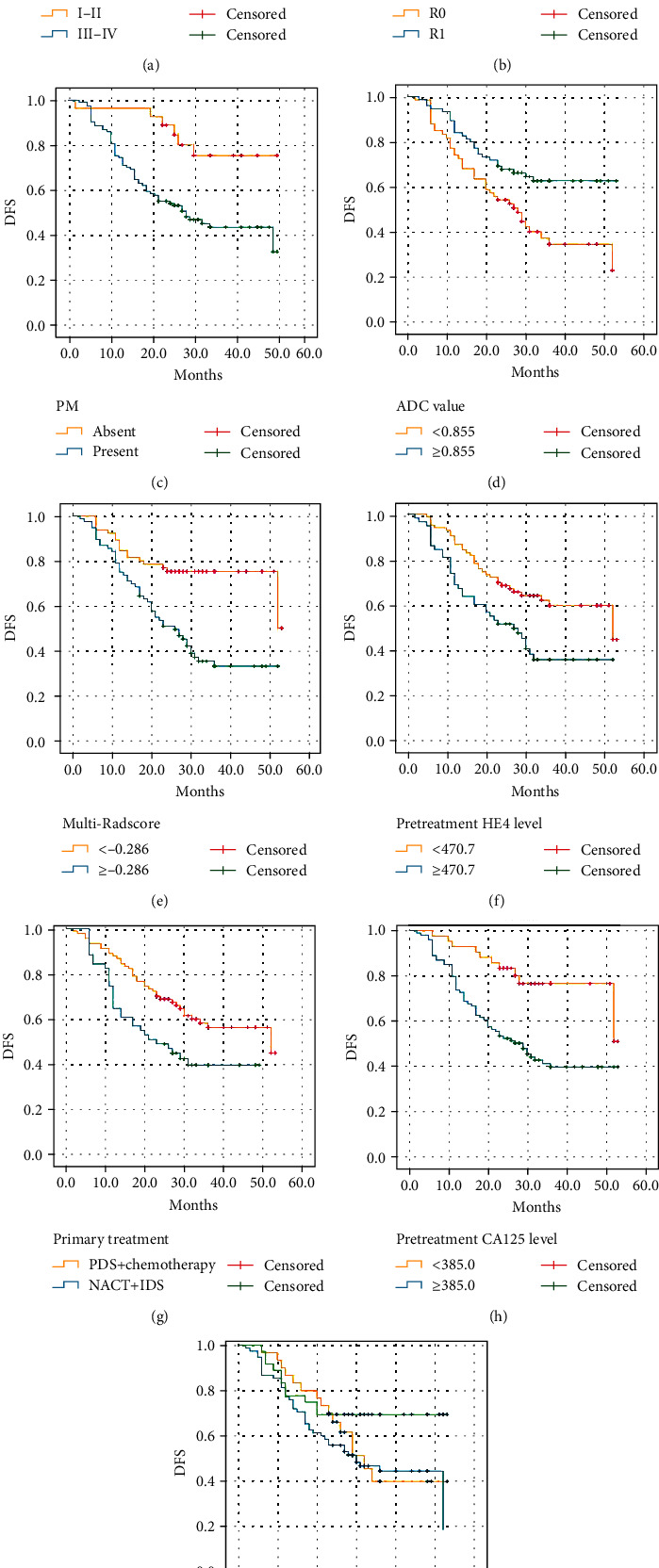
DFS curves of patients with HGSOC in the overall cohort. (a) FIGO stage I-II vs. III-IV, *P* = 0.004. (b) Residual tumour status R0 vs. R1, *P* < 0.001. (c) PM absent vs. present, *P* = 0.003. (d) ADC ≤ 0.855 vs. >0.855, *P* = 0.005. (e) Multi-Radscore < −0.286 vs. ≥-0.286, *P* < 0.001. (f) Pretreatment HE4 < 470.7 vs. ≥470.7, *P* = 0.005. (g) Primary treatment PDS+chemotherapy vs. NACT+IDS, *P* = 0.013. (h) Pretreatment CA125 < 385.0 vs. ≥385.0, *P* = 0.001. (i) Tumour composition cystic vs. solid vs. solid-cystic, *P* = 0.103.

The results of Kaplan–Meier survival analysis are summarized in Table S6 and Figure 3. There was a significant difference in DFS rates between the two groups with a low or high risk of HGSOC recurrence.

Then, Cox regression analysis showed that residual tumour status and multi-Radscore were independent risk factors for recurrence of HGSOC. The results of the specific stratification factors are shown in Table 3.

## 4. Discussion

HGSOC has obvious histological heterogeneity, exhibiting a variety of growth patterns, including papillary, solid, and glandular, and it often contains regions of cysts, necrosis, and hemorrhage [22]. Despite very high initial chemosensitivity and a complete clinical response, the majority of patients relapse within the first 5 years and progressively develop resistance to various chemotherapeutic treatments [4]. Recent studies have shown that postoperative residual tumour status plays an important role in tumour recurrence [5, 23, 24]. However, the residual tumour status can only be obtained postoperatively.

As a routine preoperative imaging examination of OC, FS-T2WI, T1WI+C, and DWI sequences of MRI can provide information about the morphology, microvascular permeability, and cellular structure of tumours. Radiomics analysis, the process of converting digital medical images into mineable high-dimensional data, is motivated by the concept that biomedical images contain information that reflects the underlying pathophysiology and that these relationships can be revealed via quantitative image analyses [25]. Therefore, a combination of clinical and radiomics features may reveal prognostic information for HGSOC.

To the best of our knowledge, there are only limited studies on MRI-based radiomics markers in ovarian cancer. Therefore, the initial objective of this study was to develop and validate a fusion model (Nomogram) based on MRI multisequence signatures and clinical factors to predict the recurrence risk in patients with HGSOC.

In this study, high levels of CA125 and HE4, lower ADC values, tumour residual state (R1), advanced FIGO stage (III-IV), different tumour compositions, PM state, and primary treatment (more with NACT and IDS) were associated with shorter DFS. The level of CA125 is widely used in screening, diagnosis, monitoring of efficacy during chemotherapy, and follow-up management of OC [26, 27]. A previous study found that glycogen CA125 of tumour cells binds to natural killer (NK) cells and is conducive to immune evasion [26], which can explain why higher CA125 levels are associated with shorter DFS. However, the high false-positive rate of CA125 may place an unnecessary psychological and therapeutic burden on women without OC. Another important monitoring indicator is serum HE4. HE4 has received authorization from the FDA to monitor disease progression or recurrence of OC [28, 29]. The combination of CA125 and HE4 can maintain high specificity and improve the sensitivity of diagnosis [30]; therefore, the FDA recently approved the use of HE4 in conjunction with CA125 for OC follow-up [31]. To predict DFS of HGSOC, serological indicators are not enough, and other clinical factors should be considered.

The ADC is a quantitative measurement based on DWI, used to evaluate water molecule diffusion within a given tissue [18]. It is influenced mainly by tissue cellularity and the integrity of the cell membranes. Lower ADC values represent a denser cellular structure and are widely and consistently used for differentiating benign and malignant diseases and in disease risk stratification. In this study, the ADC value of the recurrence group was lower than that of the nonrecurrence group, further verifying the role of ADC in clinical practice.

Similar to previous studies [1, 5, 32, 33], our study found that patients with advanced HGSOC were overwhelmingly associated with peritoneal metastases (PM) and thus were more likely to have tumour residue after PDS; all factors were associated with a shorter DFS. The presence of residual tumour is an independent risk factor for recurrence. To date, few researchers have considered the effect of tumour composition on prognosis. Tumours can be classified as having cystic, solid, and cystic-solid components. In this study, different tumour components were also found to have an effect on tumour recurrence, although they were not independent predictors in multivariate analysis. By observing the tumour images of the recurrence group and the nonrecurrence group, we found that solid tumours accounted for the majority of the two groups, while cystic tumours accounted for the lowest proportion in the recurrence group. It was speculated that tumours with more solid components were more aggressive and more likely to lead to disease recurrence.

In addition, the initial treatment strategy also strongly affects disease recurrence. According to the guidelines, the standard treatment for HGSOC is PDS and postoperative adjuvant chemotherapy. However, due to the late discovery of ovarian cancer and its extensive metastasis at the time of diagnosis, the surgical scope is wide, and the trauma is large. The need for the combined efforts of multidisciplinary experts to completely remove the tumour often leads to poor postoperative recovery and even accelerates the occurrence of death of patients due to the significant trauma. Therefore, NACT followed by IDS has been proposed for the management of advanced HGSOC to increase the rate of complete cytoreductive surgery and to reduce the postoperative complication rate and mortality [4, 34]. Previous studies have confirmed that PFS and OS improved significantly with NACT and IDS in patients diagnosed with FIGO IV disease, while PFS with PDS was superior in patients with FIGO III with small extrapelvic metastases [35]. Therefore, when selecting treatment strategies, we must consider not only the risk of perioperative morbidity and the possibility of metastasis from a residual-free tumour but also FIGO staging and the extent of metastatic disease. Neither of the two investigated procedures has proven to be superior in terms of OS and PFS for the treatment of advanced HGSOC. The results of our study are consistent with most studies showing that the most important prognostic factor is the absence of residual disease [36].

Two other clinical factors have to be mentioned, the neutrophil-to-lymphocyte ratio (NLR) and plasma fibrinogen. The NLR, a representative indicator of inflammatory status, has been reported as a prognostic marker for many solid malignancies, including OC [37]. Plasma fibrinogen is a liver-produced protein converted from fibrin by activated thrombin and is increased in the presence of malignant tumours or systemic inflammation [38]. Inflammation is known to play an important role in the development and progression of cancer. So, the F-NLR, a combination of NLR and fibrinogen, has been proposed as a prognostic marker in several tumours. However, in this study, no statistical differences in NLR and fibrinogen were found between the recurrence group and the nonrecurrence group, which was different from the results of Yang et al. Yang et al. [38] showed that F-NLR was an independent prognostic factor for EOC survival. The later the clinical stage of FIGO, the higher the lymph node metastasis and CA-125 level, the higher the F-NLR score, and the shorter the DFS and OS. Marchetti et al. [39] also confirmed that the F-NLR could predict tumour burden, platinum resistance, and negative prognostic impact on PFS. Neutrophil and lymphocyte counts are nonspecific parameters based on inflammation and are strongly influenced by infection, inflammation, and drugs [39], which may account for the different findings.

By using the least absolute shrinkage and selection operator (LASSO) by unsupervised analysis, seven radiomics features were extracted. They are DWI-based wavelet-LLH-first order-kurtosis and original-shape-flatness; T1WI+C-based wavelet-LHL-glcm-correlation and original-shape-flatness; and FS-T2WI-based wavelet-LLH-glcm-idn, original-glcm-MCC, and original-glszm-small area low grey level emphasis. They represent the kurtosis, shape, correlation, and texture of the tumour and are not visible to the naked eye of radiologists. The AUC of the clinical model was lower than that of the multi-Radscore model, and the AUC of the combined model was higher than that of either the clinical model or multi-Radscore model (*P* = 0.044 and 0.011, DeLong test). These results indicated that the combined model could be used to assess the added value of radiomics features over clinical risk factors in HGSOC patients; that is, radiomics features could be used to improve the diagnostic value of predicting relapse of HGSOC patients.

Wei et al. [6] and Chen et al. [40] utilized a radiomics approach to predict recurrence in patients with HGSOC based on preoperative CT and proved that the combined model has higher predictive efficiency, which is consistent with the present study. The difference is that our study used an mMRI-based radiomics approach, obtained more sufficient tumour information, and confirmed that the diagnostic efficacy of the multisequence radiomics model was better than that of a single sequence model. Although residual tumour status was the only independent risk factor confirmed in this study, the other factors were included in the nomograms considering the important significance of serological indicators, FIGO stage, and other clinical factors. Nomograms show the weights of these factors, and their combined total risk is the probability of relapse of HGSOC patients. Considering the role of clinical and radiomics signatures, it can help gynecologists change treatment strategies early and improve patient management.

ROC curves were drawn for clinical risk factors and the Radscore in the combined model to determine the optimal cut-off value, and patients were divided into a high-risk group and a low-risk group. Kaplan–Meier curves can be used to significantly differentiate DFS between high-risk and low-risk relapse groups based on the combined model. DFS decreased over time in both the high-risk and low-risk groups, while DFS decreased more rapidly in the high-risk groups. Therefore, for high-risk individuals, the follow-up interval should be shortened, and education about the signs and symptoms of recurrence (such as pelvic pain, abdominal bloating, early satiety, obstruction, weight loss, and fatigue) should be provided. At the same time, physical examination and identification of CA125 levels or corresponding tumour markers are recommended at each follow-up. In addition, according to the NCCN guidelines [41], molecular tumour testing is recommended before starting treatment for persistent/recurrent disease so that clinicians can tailor drug treatment plans for individual patients, and patients are encouraged to participate in clinical trials as early as possible. Thus, the combinatorial model confirms the value of radiomics approaches in better understanding HGSOC recurrence.

Kaplan–Meier survival analysis showed that higher FIGO stage, postoperative residual tumour status, peritoneal metastases, NACT+IDS treatment strategy, higher CA125 and HE4 levels, and lower ADC and Radscore values predicted shorter DFS, with statistically significant differences. There was no statistically significant difference between different tumour components in predicting DFS. Further Cox survival analysis confirmed that postoperative residual tumour status and higher multi-Radscore were more likely to lead to early postoperative recurrence and a shorter DFS.

In this study, BRCA mutations were not studied in depth due to the lack of relevant data. Genetic counselling and testing rates for OC are less than 7% [42]. Alsop et al. [42] have shown that BRCA mutations are associated with longer survival after the diagnosis of OC and an overall favourable response to platinum-based therapy. BRCA mutation status was an independent predictor of OS and PFS improvement in multivariate analysis. However, Marchetti et al. [37] found evidence that BRCA mutation of OC is not “a disease” with unique and favourable survival outcomes. Even in the presence of BRCA mutations, the prognosis can be determined by other factors, such as the NLR. In addition, the reference value of NLR, as well as the correlation between BRCA and F-NLR, has not yet been uniformly defined and requires further study.

There are still many limitations in this research. First, our study only considered HGSOC and did not discuss other subtypes of ovarian cancer. In addition, in this retrospective study, the stage of the patients was relatively high, and the sample size was limited, which would introduce a certain bias into the results. Second, we did not carry out external validation, which is one of the key factors to be considered in future research. Finally, as with any radiomics study, manual profiling errors are unavoidable.

In conclusion, our study analysed in detail the influence of preoperative, postoperative, and clinical factors along with imaging features on the early recurrence of HGSOC patients and constructed a nomogram combining clinical factors and radiomics models for predicting the early recurrence of HGSOC patients. Our results indicate that the proposed radiomics nomogram can be used to improve the accuracy of preoperatively identifying patients with HGSOC who have a high risk of recurrence. Our study provides a new tool for the early prognostic assessment of patients and the development of personalized treatment.

## Figures and Tables

**Table 1:** Clinical and pathological characteristics of patients.

	Overall (*n* = 141)		
	Nonrecurrence (*n* = 76)	Recurrence (*n* = 65)	*P* value
Age (y), M (IQR)	53.0 (48.3, 60.8)	55.0 (49.0, 62.5)	0.290
CA125, M (IQR)	480.1 (220.4, 1062.3)	917.7 (564.5, 2309.0)	0.001
HE4, M (IQR)	293.5 (175.4, 589.5)	499.1 (271.2, 1059.5)	0.001
ADC value, M (IQR)	0.90 (0.77. 0.99)	0.83 (0.73, 0.92)	0.011
Fibrinogen, M (IQR)	4.17 (3.54, 4.90)	4.17 (3.22, 4.71)	0.565
NLR, M (IQR)	2.98 (2.04, 3.82)	3.05 (1.91, 4.48)	0.434
Residual tumour status, *n* (%)			<0.001
R0	51 (39.9)	23 (34.1)	
R1	25 (36.1)	42 (30.9)	
Tumour location, *n* (%)			0.211
Unilateral	36 (32.3)	24 (27.7)	
Bilateral	40 (43.7)	41 (37.3)	
FIGO, *n* (%)			0.004
I-II	16 [4]	3 (8.8)	
III-IV	60 (65.8)	62 (56.2)	
Tumour composition, *n* (%)			0.042
Cystic	17 (16.2)	13 (13.8)	
Solid	46 (40.4)	29 (34.6)	
Solid-cystic	13 (19.4)	23 (16.6)	
Hemorrhage, *n* (%)			0.880
+	10 (9.7)	8 (8.3)	
-	66 (66.3)	57 (56.7)	
ER, *n* (%)			0.656
+	65 (63.6)	53 (54.4)	
-	4 (5.4)	6 (4.6)	
+/-	7 (7.0)	6 (6.0)	
PR, *n* (%)			0.649
+	43 (44.2)	39 (37.8)	
-	26 (23.7)	18 (20.3)	
+/-	7 (8.1)	8 (6.9)	
PM, *n* (%)			0.006
+	55 (61.4)	59 (52.6)	
-	21 (14.6)	6 (12.4)	
Primary treatment, *n* (%)			0.023
NACT+IDS	21 (27.5)	30 (23.5)	
PDS+chemotherapy	55 (48.5)	35 (41.5)	
Ki-67 PI, *n* (%)			0.629
High	61 (59.8)	50 (51.2)	
Low	15 (16.2)	15 (13.8)	

M: median; IQR: interquartile spacing; CA125: carbohydrate antigen 125; HE4: human epididymis protein 4; ADC: apparent diffusion coefficient; NLR: neutrophil-to-lymphocyte ratio; FIGO: International Federation of Gynecology and Obstetrics; ER: estrogen receptor; PR: progesterone receptor; PM: peritoneal metastasis; NACT: new adjuvant chemotherapy treatment; IDS: interval debulking surgery; PDS: primary debulking surgery; PI: proliferation index.

**Table 2:** Performance of the clinical model, radiomics model, and clinical-radiomics model in the training cohort and test cohort.

	Training cohort						Validation cohort					
	AUC	ACC	SEN	SPE	PPV	NPV	AUC	ACC	SEN	SPE	PPV	NPV
Clinical model	0.76 (0.68, 0.84)	0.74	0.59	0.89	0.84	0.68	0.67 (0.53, 0.81)	0.59	0.48	0.70	0.61	0.57
DWI radiomics	0.76 (0.68, 0.83)	0.69	0.60	0.77	0.73	0.66	0.74 (0.61, 0.85)	0.65	0.44	0.87	0.77	0.61
T1WI+C radiomics	0.73 (0.64, 0.80)	0.67	0.66	0.68	0.67	0.67	0.72 (0.58, 0.83)	0.70	0.52	0.87	0.80	0.65
FS-T2WI radiomics	0.72 (0.64, 0.80)	0.69	0.83	0.55	0.65	0.76	0.70 (0.56, 0.82)	0.63	0.65	0.61	0.63	0.64
Multiradiomics	0.78 (0.71, 0.86)	0.75	0.64	0.85	0.81	0.70	0.74 (0.61, 0.86)	0.67	0.52	0.83	0.75	0.63
Nomogram	0.83 (0.77, 0.90)	0.78	0.73	0.81	0.77	0.78	0.78 (0.65, 0.90)	0.77	0.80	0.74	0.73	0.81

AUC: area under the curve; ACC: accuracy; SEN: sensitivity; SPE: specificity; PPV: positive predictive value; NPV: negative predictive value; DWI: diffusion-weighted imaging; T1WI + C: contrast-enhanced T1-weighted imaging; FS-T2WI: fat-suppressed T2-wighted imaging.

**Table 3:** The result of Cox regression survival analysis.

Factors	HR	95% CI	*P* value
FIGO stage (I-II vs. III-IV)	1.209	0.395-3.704	0.740
Residual tumour status (R0 vs. R1)	2.711	1.283-5.728	0.009
PM (absent vs. present)	1.211	0.446-3.288	0.707
Primary treatment (PDS+chemotherapy vs. NACT+IDS)	0.694	0.337-1.427	0.321
Primary treatment CA125 level (<385.0 vs. ≥385.0)	1.581	0.786-3.181	0.199
Primary treatment HE4 level (<470.7 vs. ≥470.7)	1.175	0.681-2.026	0.563
ADC value (<0.855 vs. ≥0.855)	0.802	0.485-1.328	0.392
Tumour composition (cystic, solid, and solid-cystic)	0.808	0.555-1.176	0.265
Multi-Radscore (<-0.286 vs. ≥-0.286)	2.302	1.177-4.503	0.015

FIGO: International Federation of Gynecology and Obstetrics; PM: peritoneal metastasis; PDS: primary debulking surgery; NACT: new adjuvant chemotherapy treatment; IDS: interval debulking surgery; ADC: apparent diffusion coefficient; HR: hazard ratio; CI: confidence interval.

## Data Availability

The raw data supporting the conclusions of this article will be made available by the authors, without undue reservation.

## Supplementary Materials

Supplementary Materials Supplementary Table 1: MRI protocol parameters. Supplementary Table 2: texture features in this study. Supplementary Table 3: the results of the multivariate analysis. Supplementary Table 4: clinical characteristics of patients in training and validation cohorts. Supplementary Table 5: the results of radiomics features selection in the training cohort. Supplementary Table 6: Kaplan-Meier survival analysis of patients in training group. Supplementary Figure 1: flowchart of the inclusion and exclusion criteria for the study. Supplementary Figure 2: the detailed description of the radiomics images preprocessing. Supplementary Figure 3: the ROC, calibration curve, and DCA of the each model in validation cohort.
