# Supplementary material for: A Nomogram Combining MRI Multisequence Radiomics and Clinical Factors for Predicting Recurrence of High-Grade Serous Ovarian Carcinoma
Source: J Oncol. 2022 May 4;2022:1716268. doi: 10.1155/2022/1716268 (PMC9095390; doi:10.1155/2022/1716268)
Supplement: Supplementary Materials — Supplementary Table 1: MRI protocol parameters. Supplementary Table 2: texture features in this study. Supplementary Table 3: the results of the multivariate analysis. Supplementary Table 4: clinical characteristics of patients in training and validation cohorts. Supplementary Table 5: the results of radiomics features selection in the training cohort. Supplementary Table 6: Kaplan-Meier survival analysis of patients in training group. Supplementary Figure 1: flowchart of the inclusion and exclusion criteria for the study. Supplementary Figure 2: the detailed description of the radiomics images preprocessing. Supplementary Figure 3: the ROC, calibration curve, and DCA of the each model in validation cohort. [file 1716268.f1.zip › 1716268.f1/Supplementary material (1).docx]

**Supplementary material**

**Table S1** MRI protocol parameters.

| **Sequences** | **TE (ms)** | **TR (ms)** | **Freq × phase** | **Nex** | **FOV** | **Slice thickness** | **Interval** | **Flip angle** |
| --- | --- | --- | --- | --- | --- | --- | --- | --- |
| FS T2WI | 72.5 | 5,000 | 320 × 256 | 2 | 24 × 24 | 6 | 2 | 90° |
| T2WI | 72.5 | 4,600 | 320 × 256 | 2 | 24 × 24 | 6 | 2 | 90° |
| Osag T2WI | 72 | 4,500 | 320 × 320 | 2 | 28 × 28 | 4 | 1 | 90° |
| T1WI | 7.5 | 500 | 352 × 192 | 2 | 32 × 32 | 6 | 2 | 90° |
| DWI (b= 0, 1,000 s/mm^2^) | / | 5,000 | 96 × 130 | 6 | 32 × 32 | 6 | 2 | 90° |
| Oax LAVA-FLEX | 1.4 | 5.8 | 320 × 224 | 1 | 34 × 31 | 4 | 0 | 15° |
| Osag LAVA-FLEX | 1.3 | 6.8 | 268 × 224 | 1 | 28 × 25 | 4 | 0 | 15° |

**Table S2** Texture features in this study.

| Method | Features |
| --- | --- |
| First order | Energy, Total Energy, Entropy, Minimum, 10th Percentile, 90th Percentile, Maximum, Mean, Median, Interquartile Range (IR), Range, Mean Absolute Deviation (MAD), Robust Mean Absolute Deviation (RMAD), Root Mean Squared (RMS), Skewness, Kurtosis, Variance, Uniformity |
| Shape Features (2D) | Mesh Surface, Pixel Surface, Perimeter, Perimeter to Surface ratio, Sphericity, Spherical Disproportion, Maximum 2D diameter, Major Axis Length, Minor Axis Length, Elongation |
| Shape Features (3D) | Mesh Volume, Voxel Volume, Surface Area, Surface Area to Volume ratio, Sphericity, Compactness 1, Compactness 2, Spherical Disproportion, Maximum 3D diameter, Maximum 2D diameter (Slice), Maximum 2D diameter (Column), Maximum 2D diameter (Row), Major Axis Length, Minor Axis Length, Least Axis Length, Elongation, Flatness |
| Gray Level Co-occurrence Matrix (GLCM) | Autocorrelation, Joint Average, Cluster Prominence, Cluster Shade, Cluster Tendency, Contrast, Correlation, Difference Average, Difference Entropy, Difference Variance, Joint Energy, Joint Entropy, Informational Measure of Correlation (IMC) 1, Informational Measure of Correlation (IMC) 2, Inverse Difference Moment (IDM), Maximal Correlation Coefficient (MCC), Inverse Difference Moment Normalized (IDMN), Inverse Difference (ID), Inverse Difference Normalized (IDN), Inverse Variance, Maximum Probability, Sum Average, Sum Entropy, Sum of Squares |
| Gray Level Run Length Matrix (GLRLM) | Short Run Emphasis (SRE), Long Run Emphasis (LRE), Gray Level Non-Uniformity (GLN), Gray Level Non-Uniformity Normalized (GLNN), Run Length Non-Uniformity (RLN), Run Length Non-Uniformity Normalized (RLNN), Run Percentage (RP), Gray Level Variance (GLV), Run Variance (RV), Run Entropy (RE), Low Gray Level Run Emphasis (LGLRE), High Gray Level Run Emphasis (HGLRE), Short Run Low Gray Level Emphasis (SRLGLE), Short Run High Gray Level Emphasis (SRHGLE), Long Run Low Gray Level Emphasis (LRLGLE), Long Run High Gray Level Emphasis (LRHGLE) |
| Gray Level Size Zone Matrix (GLSZM) | Small Area Emphasis (SAE), Large Area Emphasis (LAE), Gray Level Non-Uniformity (GLN), Gray Level Non-Uniformity Normalized (GLNN), Size-Zone Non-Uniformity (SZN), Size-Zone Non-Uniformity Normalized (SZNN), Zone Percentage (ZP), Gray Level Variance (GLV), Zone Variance (ZV), Zone Entropy (ZE), Low Gray Level Zone Emphasis (LGLZE), High Gray Level Zone Emphasis (HGLZE), Small Area Low Gray Level Emphasis (SALGLE), Large Area Low Gray Level Emphasis (LALGLE), Large Area High Gray Level Emphasis (LAHGLE) |
| Neighbouring Gray Tone Difference Matrix (NGTDM) | Coarseness, Contrast, Busyness, Complexity, Strength |
| Gray Level Dependence Matrix (GLDM) | Small Dependence Emphasis (SDE), Large Dependence Emphasis (LDE), Gray Level Non-Uniformity (GLN), Dependence Non-Uniformity (DN), Dependence Non-Uniformity Normalized (DNN), Gray Level Variance (GLV), Dependence Variance (DV), Dependence Entropy (DE), Low Gray Level Emphasis (LGLE), High Gray Level Emphasis (HGLE), Small Dependence Low Gray Level Emphasis (SDLGLE), Small Dependence High Gray Level Emphasis (SDHGLE), Large Dependence Low Gray Level Emphasis (LDLGLE), Large Dependence High Gray Level Emphasis (LDHGLE) |

**Table S3** The results of the multivariate analysis.

|  | B value | *P* value | OR | 95% CI for OR |
| --- | --- | --- | --- | --- |
| Pretreatment CA125 | <0.001 | 0.178 | 1.000 | 1.000-1.000 |
| Pretreatment HE4 | 0.001 | 0.238 | 1.001 | 1.000-1.001 |
| ADC value | -1.203 | 0.334 | 0.300 | 0.026-3.440 |
| FIGO stage | 0.862 | 0.275 | 2.368 | 0.503-11.139 |
| Tumor composition | 0.215 | 0.452 | 1.240 | 0.707-2.176 |
| PM | 0.503 | 0.414 | 1.654 | 0.495-5.529 |
| Primary treatment | -1.086 | 0.111 | 0.338 | 0.089-1.285 |
| Residual tumor status | 1.505 | 0.021 | 4.506 | 1.252-16.216 |

*CA125* carbohydrate antigen 125, *HE4* human epididymis protein 4, *ADC* apparent diffusion coefficient, *FIGO* international federation of gynecology and obstetrics, *PM* peritoneal metastasis, *OR* odds ratio

**Table S4** Clinical characteristics of patients in training and validation cohorts.

|  | Training Cohort (n=98) | | | Validation Cohort (n=43) | | |
| --- | --- | --- | --- | --- | --- | --- |
|  | Non-recurrence (n =53) | Recurrence (n=45) | *P* value | Non-recurrence (n =23) | Recurrence (n=20) | *P* value |
| Age (y), M (IQR) | 53.0 (49.5, 62.0) | 55.0 (49.5, 61.0) | 0.484 | 53.0 (48.0, 60.0) | 55.5 (48.3, 65.0) | 0.262 |
| CA125, M (IQR) | 598.8 (222.8, 1056.5) | 970.1 (499.9, 2237.0) | 0.005 | 371.7 (192.0, 1483.0) | 878.1 (638.5, 2669.8) | 0.040 |
| HE4, M (IQR) | 303.6 (174.8, 721.6) | 560.0 (284.0, 1059.5) | 0.013 | 262.9 (175.3, 536.8) | 457.7 (224.6, 1280.0) | 0.039 |
| ADC value, M (IQR) | 0.90 (0.80, 1.01) | 0.81 (0.70. 0.90) | 0.003 | 0.90 (0.77, 0.99) | 0.83 (0.73, 0.92) | 0.105 |
| Fibrinogen, M (IQR) | 4.17 (3.38, 4.63) | 4.17 (3.37, 4.79) | 0.770 | 4.19 (3.77, 5.38) | 4.07 (2.91, 4.63) | 0.201 |
| NLR, M (IQR) | 2.75 (1.74, 3.45) | 3.16 (2.17, 4.63) | 0.067 | 3.20 (2.40, 4.01) | 2.41 (1.83, 3.75) | 0.141 |
| Residual tumor status, n (%) |  |  | 0.002^a^ |  |  | 0.025^a^ |
| R0 | 34 (26.5) | 15 (22.5) |  | 17 (13.4) | 8 (11.6) |  |
| R1 | 19 (26.5) | 30 (22.5) |  | 6 (9.6) | 12 (8.4) |  |
| Tumor Location, n (%) |  |  | 0.761^a^ |  |  | 0.069^a^ |
| Unilateral | 24 (23.3) | 19 (19.7) |  | 12 (9.1) | 5 (7.9) |  |
| Bilateral | 29 (29.7) | 26 (25.3) |  | 11 (13.9) | 15 (12.1) |  |
| FIGO, n (%) |  |  | 0.047^a^ |  |  | 0.082^b^ |
| Ⅰ-Ⅱ | 11 (7.6) | 3 (6.4) |  | 5 (2.7) | 0 (2.3) |  |
| Ⅲ-Ⅳ | 42 (45.4) | 42 (38.6) |  | 18 (20.3) | 20 (17.7) |  |
| Tumor composition, n (%) |  |  | 0.307^a^ |  |  | 0.061^c^ |
| Cystic | 11 (11.4) | 10 (9.6) |  | 6 (4.8) | 3 (4.2) |  |
| Solid | 32 (28.7) | 21 (24.3) |  | 14 (11.8) | 8 (10.2) |  |
| Solid-cystic | 10 (13.0) | 14 (11.0) |  | 3 (6.4) | 9 (5.6) |  |
| Hemorrhage, n (%) |  |  | 0.103^b^ |  |  | 0.162^b^ |
| + | 8 (5.4) | 2 (4.6) |  | 2 (4.3) | 6 (3.7) |  |
| - | 45 (47.6) | 43 (40.4) |  | 21 (18.7) | 14 (16.3) |  |
| ER, n (%) |  |  | 0.364^c^ |  |  | 0.889^c^ |
| + | 45 (43.3) | 35 (36.7) |  | 20 (20.3) | 18 (17.7) |  |
| - | 2 (3.8) | 5 (3.2) |  | 2 (1.6) | 1 (1.4) |  |
| +/- | 6 (5.9) | 5 (5.1) |  | 1 (1.1) | 1 (0.9) |  |
| PR, n (%) |  |  | 0.920^a^ |  |  | 0.525^c^ |
| + | 30 (30.3) | 26 (25.7) |  | 13 (13.9) | 13 (12.1) |  |
| - | 17 (16.2) | 13 (13.8) |  | 9 (7.5) | 5 (6.5) |  |
| +/- | 6 (6.5) | 6 (5.5) |  | 1 (1.6) | 2 (1.4) |  |
| PM, n (%) |  |  | 0.022^a^ |  |  | 0.255^b^ |
| + | 37 (41.6) | 40 (35.4) |  | 18 (19.8) | 19 (17.2) |  |
| - | 16 (11.4) | 5 (9.6) |  | 5 (3.2) | 1 (2.8) |  |
| Primary treatment, n (%) |  |  | 0.012^a^ |  |  | 0.775^a^ |
| NACT+IDS | 15 (21.1) | 24 (17.9) |  | 6 (6.4) | 6 (5.6) |  |
| PDS+chemotherapy | 38 (31.9) | 21 (27.1) |  | 17 (16.6) | 14 (14.4) |  |
| Ki-67 PI, n (%) |  |  | 0.960^a^ |  |  | 0.540^b^ |
| High | 41 (41.1) | 35 (34.9) |  | 20 (18.7) | 15 (16.3) |  |
| Low | 12 (11.9) | 10 (10.1) |  | 3 (4.3) | 5 (3.7) |  |

*M* median, *IQR* interquartile spacing, *CA125* carbohydrate antigen 125, *HE4* human epididymis protein 4, *ADC* apparent diffusion coefficient, *NLR* neutrophil-to-lymphocyte ratio, *FIGO* international federation of gynecology and obstetrics, *ER* estrogen receptor, *PR* progesterone receptor, *PM* peritoneal metastasis, *NACT* new adjuvant chemotherapy treatment, *IDS* interval debulking surgery, *PDS* primary debulking surgery, *PI* proliferation index.

**Table S5** The results of radiomic features selection in the overall cohort.

| Feature Selected | Coeffcient of LASSO-Cox | C-index (95% CI) | *P*-value |
| --- | --- | --- | --- |
| DWI-based |  |  |  |
| Wavelet LLH firstorder Kurtosis | -0.8896 | -0.889 (-1.362, -0.417) | <0.001 |
| Original shape Flatness | -0.7489 | -0.749 (-1.211, -0.287) | 0.002 |
| T1WI+C-based |  |  |  |
| Wavelet LHL glcm Correlation | -0.7670 | -0.767 (-1.209, -0.325) | 0.001 |
| Original shape Flatness | -0.4937 | -0.494 (-0.926, -0.062) | 0.025 |
| FS-T2WI-based |  |  |  |
| Wavelet LLH glcm Idn | -0.6836 | -0.684 (-1.132, -0.236) | 0.003 |
| Original glcm MCC | 0.3711 | 0.371 (-0.056, 0.798) | 0.089 |
| Original glszm Small Area Low Gray Level Emphasis | 0.3121 | 0.312 (-0.148, 0.773) | 0.184 |

*DWI* diffusion-weighted imaging, *T1WI+C* contrast-enhanced T1-weighted imaging, *FS-T2WI* fat-suppressed T2-wighted imaging, *LASSO* least absolute shrinkage and selection operator, *CI* confidence interval

**Table S6** Kaplan-Meier survival analysis of patients in training group.

| Factors | Stratification factor | Number (%) | Median RFI (months) | *P* value |
| --- | --- | --- | --- | --- |
| FIGO stage | Ⅰ-Ⅱ | 19 | NA |  |
|  | Ⅲ-Ⅳ | 122 | 30.0 | 0.004 |
| Residual tumor status | R0 | 74 | NA |  |
|  | R1 | 67 | 20.0 | <0.001 |
| PM | absent | 27 | NA |  |
|  | present | 114 | 30.0 | 0.003 |
| Primary treatment | PDS+chemotherapy | 90 | 52.0 |  |
|  | NACT+IDS | 51 | 23.0 | 0.013 |
| Pretreatment CA125 level | <385.0 U/mL | 42 | NA |  |
|  | ≥385.0 U/mL | 99 | 29.0 | 0.001 |
| Pretreatment HE4 level | <470.7 pmol/L | 83 | 52.0 |  |
|  | ≥470.7 pmol/L | 58 | 27.0 | 0.005 |
| ADC value | <0.855 | 66 | 28.0 |  |
|  | ≥0.855 | 75 | NA | 0.005 |
| Tumor composition | Cystic | 30 | 32.0 |  |
|  | Solid | 75 | 30.0 |  |
|  | Solid-cystic | 36 | NA | 0.103 |
| Multi-Radscore | <-0.286 | 65 | NA |  |
|  | ≥-0.286 | 76 | 26.0 | <0.001 |

**Figure S1** Flowchart of the inclusion and exclusion criteria for the study.

76/141 patients of HGSOC with non-recurrent disease were included

65/141 patients of HGSOC with recurrent disease were included

141 patients with HGSOC were eventually enrolled in this study

191/247 patients diagnosed with high-grade serous ovarian carcinoma (HGSOC) pathologically

247/287 patients diagnosed with serous ovarian carcinoma clinically underwent operation

Patients excluded:

14/287 patients without complete medical records

10/287 patients without surgery

16/287 patients suffered from other malignant tumors

Enter *ovarian cancer* in PACS to screen patients between 2014 and 2021, n=287

According to postoperative pathology, patients excluded:

22/247 patients diagnosed without serous ovarian carcinoma

34/247 patients diagnosed with low-grade serous ovarian carcinoma

Patients excluded:

44/191 patients without at least 18 months follow-up records

3/191 pations without complete pathological reports

3/191 patients without obvious lesion (max tumor diameter <1cm on MRI)

**Figure S2** The detailed description of the radiomics images preprocessing.


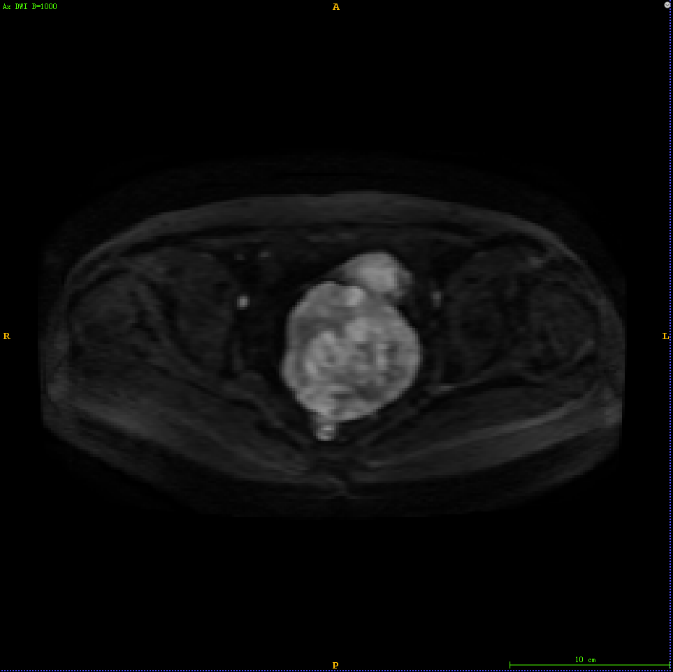

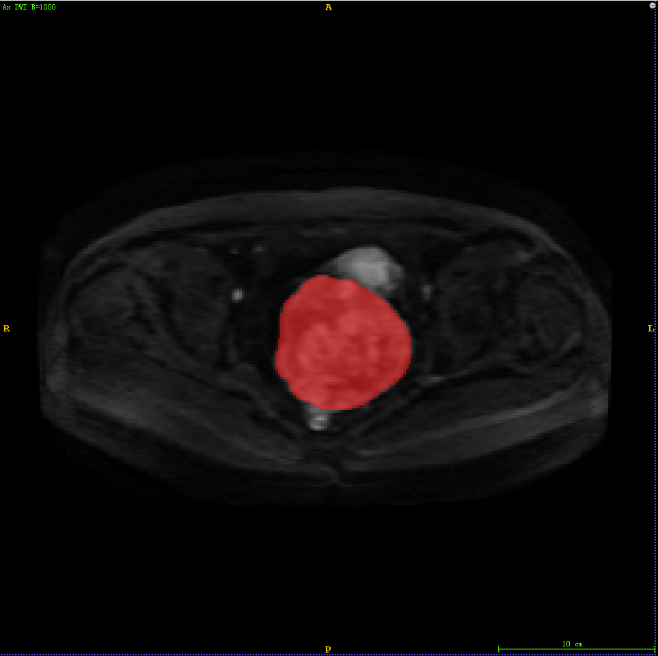


Images Acquisition


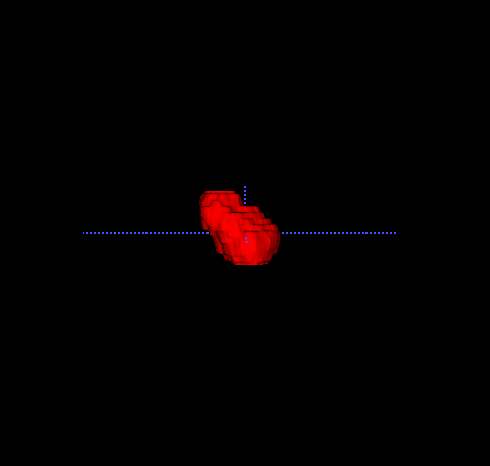


3D VOI


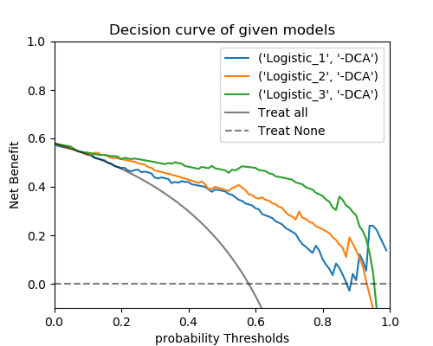

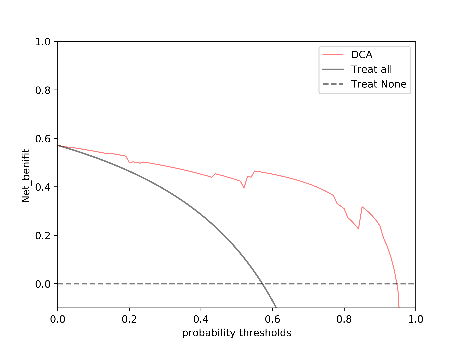


Features Extraction


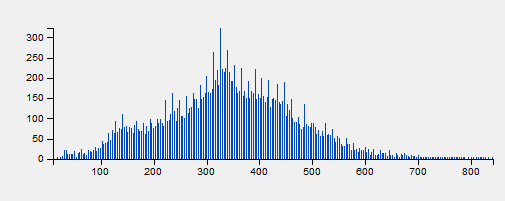


Features Selection

Features Selection

Model Construction and Performance Assessment


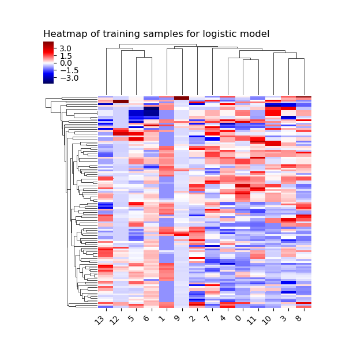

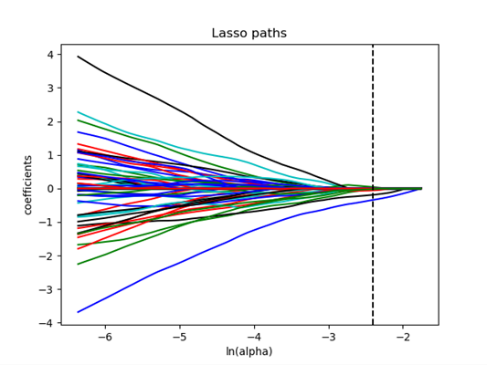

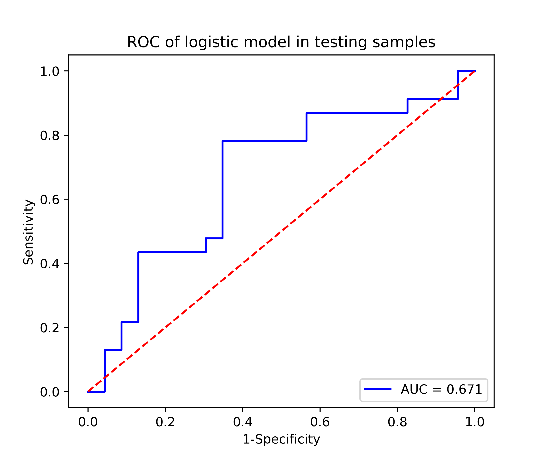


A 1


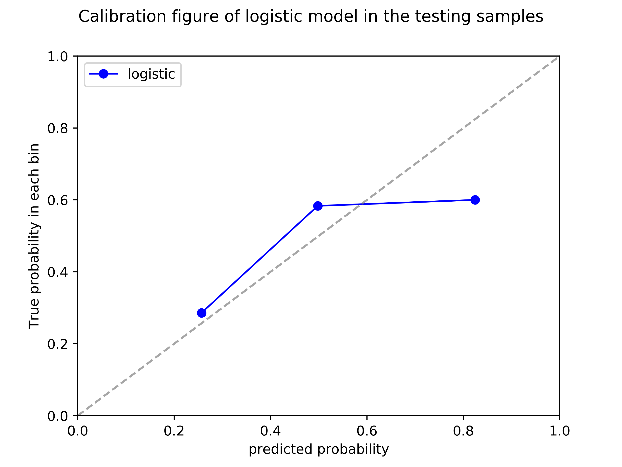


A 2


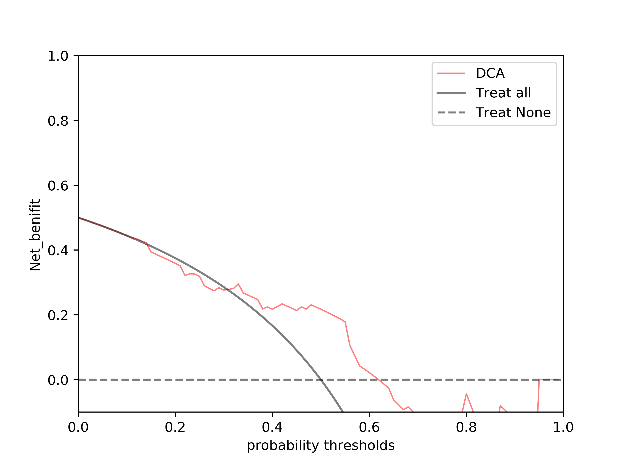


A 3


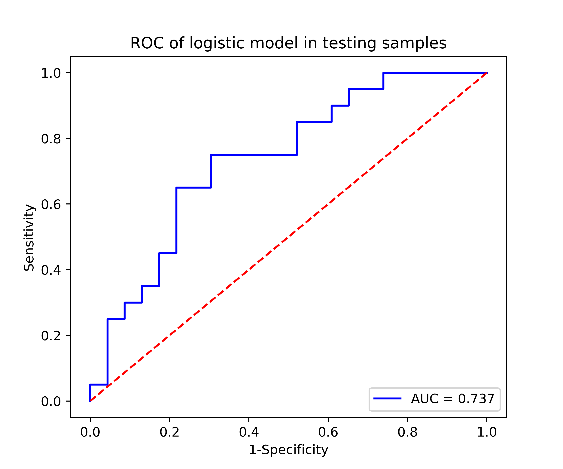


B 1


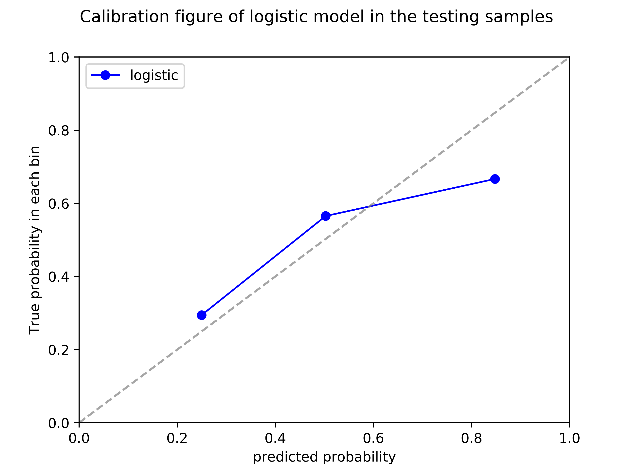


B 2


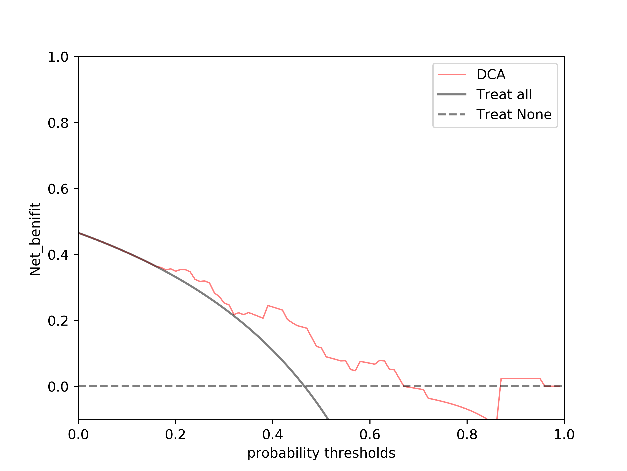


B 3


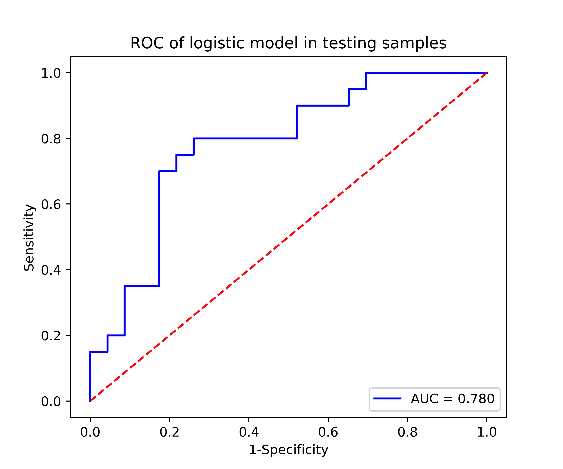


C 1


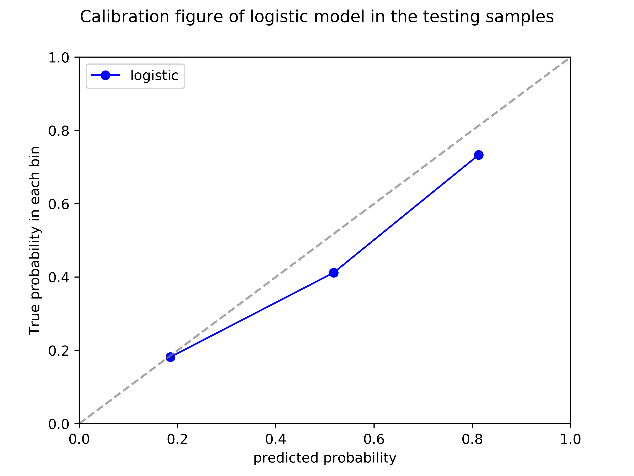


C 2


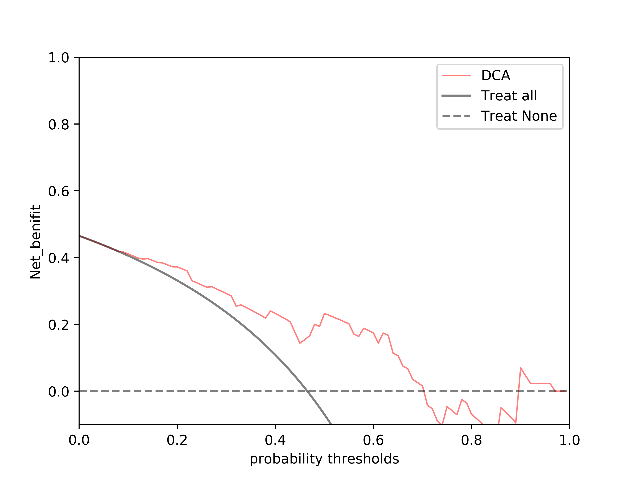


C 3

**Figure S3** (A) The ROC, Calibration curve and DCA of the clinical model in validation cohort. (B) The ROC, Calibration curve and DCA of the multi-Radscore model in validation cohort. (C) The ROC, Calibration curve and DCA of the fusion model in validation cohort.
