# Supplementary figures and images for: A Nomogram Combining MRI Multisequence Radiomics and Clinical Factors for Predicting Recurrence of High-Grade Serous Ovarian Carcinoma
Source: J Oncol. 2022 May 4;2022:1716268. doi: 10.1155/2022/1716268 (PMC9095390; doi:10.1155/2022/1716268)

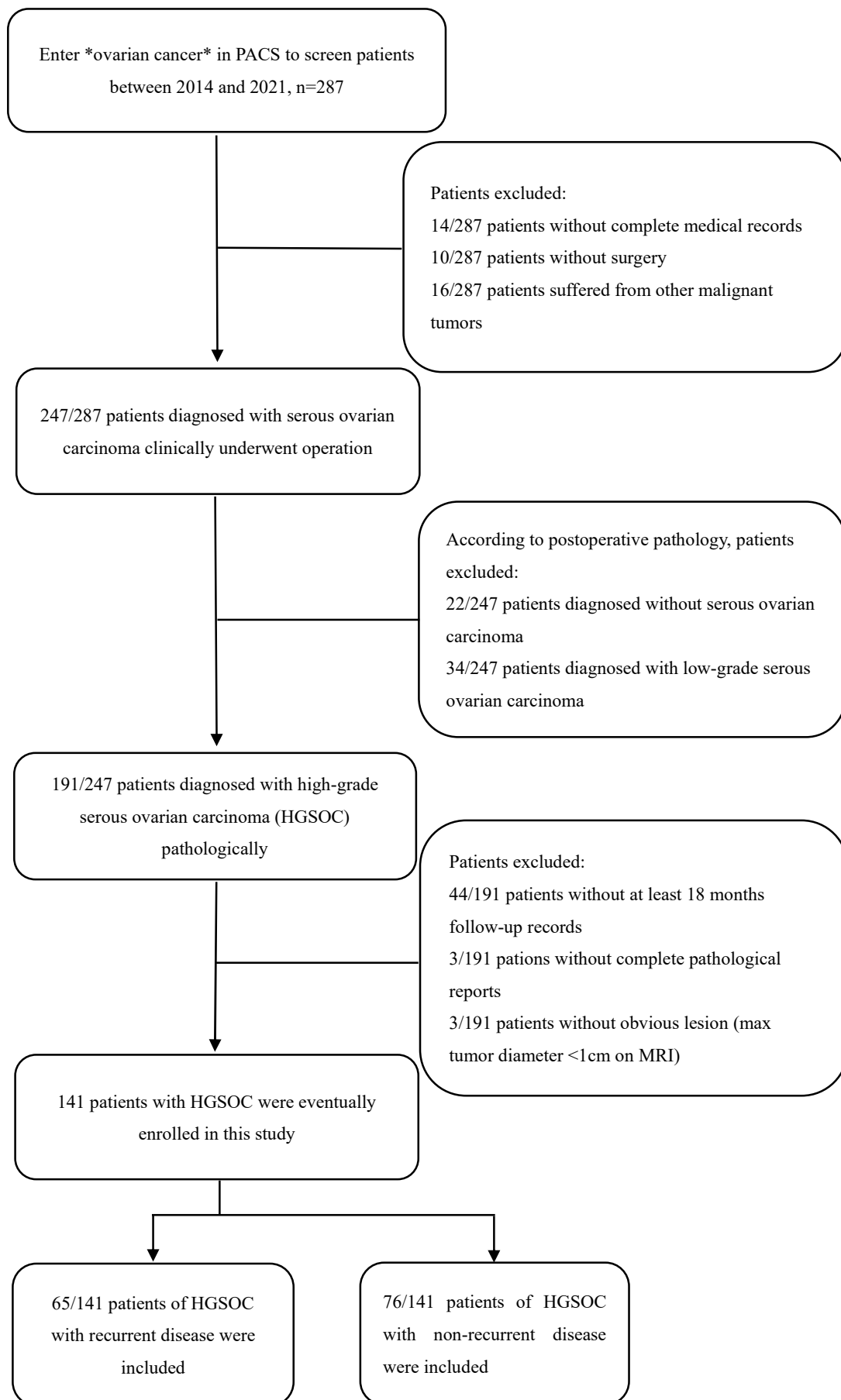

Supplement: Supplementary Materials — Supplementary Table 1: MRI protocol parameters. Supplementary Table 2: texture features in this study. Supplementary Table 3: the results of the multivariate analysis. Supplementary Table 4: clinical characteristics of patients in training and validation cohorts. Supplementary Table 5: the results of radiomics features selection in the training cohort. Supplementary Table 6: Kaplan-Meier survival analysis of patients in training group. Supplementary Figure 1: flowchart of the inclusion and exclusion criteria for the study. Supplementary Figure 2: the detailed description of the radiomics images preprocessing. Supplementary Figure 3: the ROC, calibration curve, and DCA of the each model in validation cohort. [file 1716268.f1.zip › 1716268.f1/Figure S1 Flowchart of the inclusion and exclusion criteria for the study..pdf]

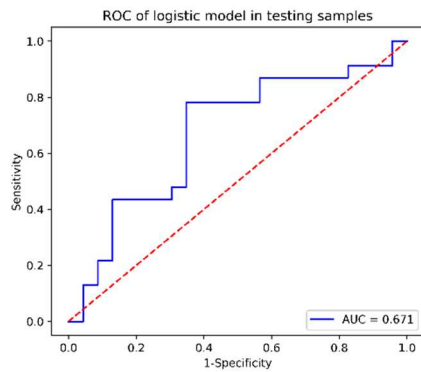

A 1

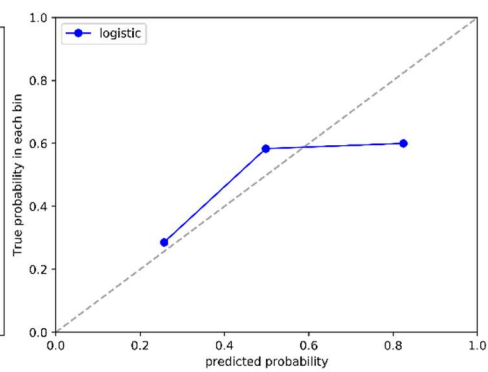

A 2

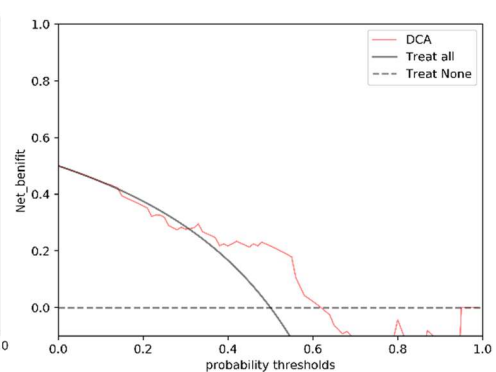

A 3

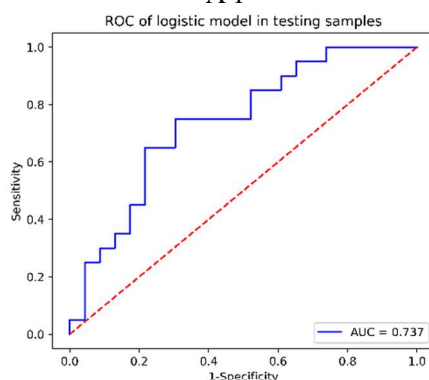

B 1

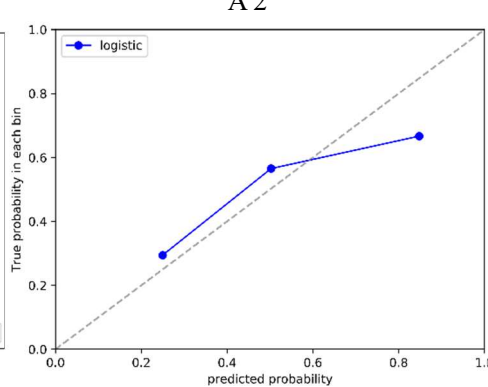

B 2

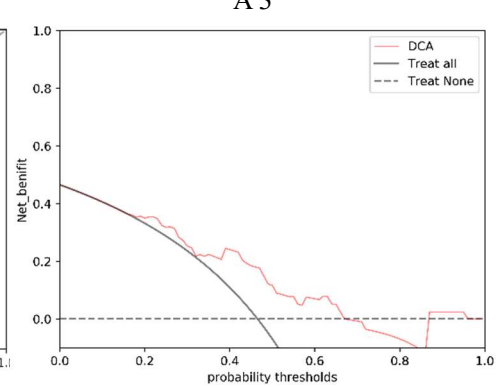

B 3

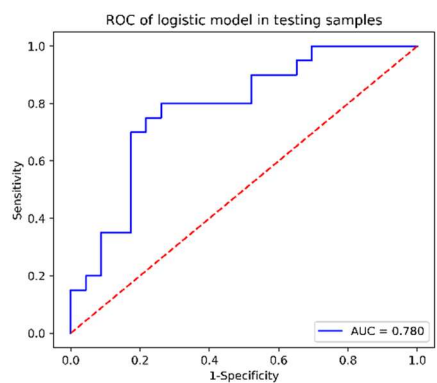

C 1

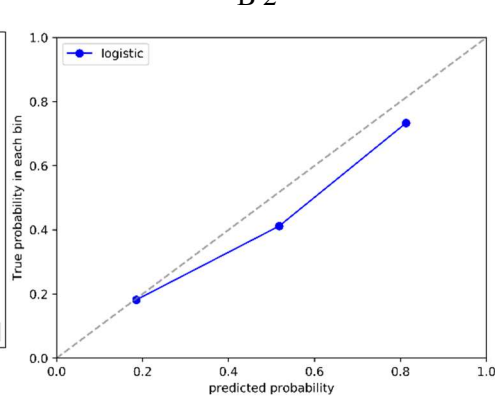

C 2

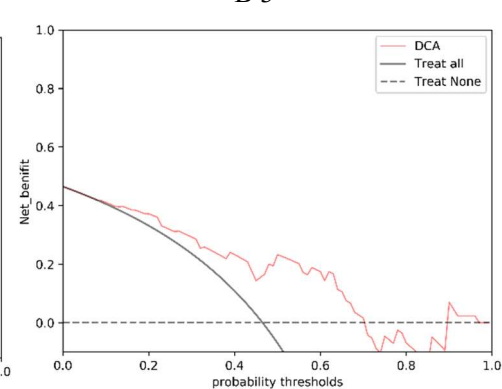

C 3

Supplement: Supplementary Materials — Supplementary Table 1: MRI protocol parameters. Supplementary Table 2: texture features in this study. Supplementary Table 3: the results of the multivariate analysis. Supplementary Table 4: clinical characteristics of patients in training and validation cohorts. Supplementary Table 5: the results of radiomics features selection in the training cohort. Supplementary Table 6: Kaplan-Meier survival analysis of patients in training group. Supplementary Figure 1: flowchart of the inclusion and exclusion criteria for the study. Supplementary Figure 2: the detailed description of the radiomics images preprocessing. Supplementary Figure 3: the ROC, calibration curve, and DCA of the each model in validation cohort. [file 1716268.f1.zip › 1716268.f1/Figure S3.pdf]
